# Technology frustration in healthcare – does it matter in staff ratings of stress, emotional exhaustion, and satisfaction with care? A cross-sectional correlational study using the job demands-resources theory

**DOI:** 10.1186/s12913-024-11906-z

**Published:** 2024-12-06

**Authors:** Maarit Wirkkala, Katarina Wijk, Agneta C. Larsson, Maria Engström

**Affiliations:** 1 Department of Caring Science, Faculty of Health and Occupational Studies, University of Gävle, Gävle, Sweden; 2 Centre for Research and Development, Region Gävleborg, Gävle, Sweden; 3 Department of Occupational Health Sciences and Psychology, Faculty of Health and Occupational Studies, University of Gävle, Gävle, Sweden; 4 Department of Public Health and Caring Sciences, Uppsala University, Uppsala, Sweden; 5 Department of Physiology and Pharmacology, Karolinska Institute, Stockholm, Sweden

**Keywords:** Burnout, Digital transformation, Emotional exhaustion, Frustration with technology, Healthcare, Health information technology, Medical informatics applications, Satisfaction with care, Stress

## Abstract

**Background:**

Health information technology has developed into a cornerstone of modern healthcare. It has changed workflows and enhanced communication, efficiency, and patient safety. However, technological development has progressed faster than research on its potential effects on care quality and the healthcare work environment. Using the Job Demand-Resources theory, this study investigated the associations between "frustration with technology" and three outcomes: stress, emotional exhaustion, and staff satisfaction with care, holding job resources and the demand workload constant.

**Method:**

A cross-sectional correlational study was conducted between January and April 2022. Healthcare staff from different professions (e.g., physicians, registered nurses, physiotherapists, licensed practical nurses) and workplaces (*n* = 417, response rate 31%) answered a survey regarding job demands and resources in the workplace, frustration with technology, stress, emotional exhaustion, and satisfaction with care. Data were analyzed with Spearman’s rank correlation coefficient, the Mann–Whitney U test, and the Kruskal–Wallis test, and multiple variables, one for each outcome, were tested with Generalized Estimated Equations models in SPSS.

**Results:**

The bivariate correlation analyses confirmed statistically significant associations between all the independent variables and the outcomes, except for the independent variable high workload. A high workload was associated with stress and emotional exhaustion but not with staff satisfaction with care. In the three GEE models, one for each outcome, higher stress was statistically significantly associated with more frustration with technology and lower scores for the variables participation in decision-making, sense of community at work, and higher workload. Higher emotional exhaustion was associated with more frustration with technology, higher workload, a lower teamwork climate, and lower growth opportunities. Lower staff satisfaction with care was associated with lower scores for the variable participation in decision-making.

**Conclusions:**

Taking other variables into account, technology frustration matters in staff ratings of stress and emotional exhaustion, but not with the satisfaction of given care. Future studies should aim to further investigate what causes technology frustration and how to mitigate it.

## Background

Over the last 25 years, health information technology (HIT) has become increasingly widespread. HIT has transformed the healthcare work environment, and users report both satisfaction and dissatisfaction with HIT [1–8]. HIT includes digital processes of storing, sharing, and analyzing health data, e.g., electronic health records (EHRs), computerized decision support systems, e-prescriptions, and digital health platforms. HIT is a means to increase patient safety and care quality [1, 4–6, 9, 10] as well as to facilitate healthcare staff communication [1, 4, 11, 12] and decision-making [13]. However, researchers from various fields have reported that information technology can increase workload [6, 14–16], frustration [1, 6, 11], stress [1, 11, 17, 18], and exhaustion [19]. When information technology contributes to staff feeling frustrated, it has been associated with stress [17, 18, 20, 21] exhaustion [10–12, 21], and care quality [22, 23].

Sweden is one of the early adopters of digital healthcare in OECD countries. Since 2006, Swedish authorities have applied a national digital health strategy, which has led to a 100% adoption of EHR in all healthcare areas [24]. It has made HIT an integrated part of healthcare [25, 26], with national regulations for healthcare staff to use digital prescriptions and EHRs. Sweden, along with Finland, is leading in patient accessibility to their EHRs with almost 100% coverage [27], including the possibility for patients to interact with healthcare professionals through different portals. However, fragmentation of patient health data persists, owing to fast innovation from different vendors targeting specific healthcare areas, and each of Sweden’s 21 regional health authorities being responsible for healthcare in their region, including choosing which HIT to use. This has led to a multitude of HIT systems, lacking interoperability, which healthcare staff navigate each day. Taken together, this makes it interesting to study technology frustration in the Swedish context.

### The Job Demands – Resources theory

According to the Job Demands-Resources (JD-R) theory, demands and resources in the workplace are associated with job strain and performance [28]. Demands and resources can be categorized as social, organizational, or individual, and a balance between them is required for staff well-being and positive organizational outcomes [29]. Workplace resources can alleviate or buffer demands [28, 30], stimulate individual growth and learning [29] and increase job performance [31]. The JD-R theory assumes that staff well-being and job performance can be negatively affected when demands are high and resources are limited [28]. High job demands and low resources have been associated with stress [28, 30], emotional exhaustion(EE) [32, 33], and burnout [28, 30, 32]. “Stress” in research can refer to either a demand (i.e., stressor) or a response to high demands (i.e., “feeling stressed”). In the latter, stress is a broader and more fluent state than burnout. The initial stress response is transient. If the demand/stressor is not alleviated over time, the stress response can become chronic, predisposing individuals to burnout [32, 34, 35]. Burnout is classified as an occupational syndrome with a symptom triad of EE, cynicism, and reduced professional efficacy [36]. EE is the core symptom of burnout [32, 37], and the burnout variable with the most robust psychometrics [38–40]; thus, it is useful for studies of work health. In healthcare research, several studies have linked high job demands and low resources to low care quality (i.e., subjective and objective measures of individual or organizational care quality) [22, 33, 41, 42], which is logical, considering care quality as a performance measurement.

### Technology frustration and staff outcomes

Frustration is a negative emotional response that occur when something obstructs a need or a goal from being fulfilled [43]. Frustration with technology can arise from many issues, such as system usability issues, poor system knowledge or training, or if the technology adds to an already heavy workload. Frustration with HIT has been associated with decreased job satisfaction [5], increased burnout [1, 10–12], and EE [44, 45] among healthcare staff. Shanafelt et al. [46] reported associations between HIT and physician burnout, indicating that technology in healthcare could have properties of work demands. These results are similar to findings from other research fields [17–21]. Heponiemi et al. [47] and Melnick et al. [48] reported that stress and burnout were associated with HIT usability problems, whereas others reported associations with information overload [49] or HIT implementation problems [10, 13]. Most studies have focused on a single HIT system, such as EHRs. Understanding the relationships between staff health, performance, and the use of a single HIT system [1, 6, 10–12] is important for evaluating a specific system. However, healthcare staff interact with multiple HITs throughout their daily work. This makes it interesting to look at HIT more generally than just a specific system. The ongoing digitization of healthcare affects several professions [3, 10–12, 46], and although studies on specific professions are important, these studies need to be supplemented and compared with studies on healthcare personnel as a group. To our knowledge, only two studies to date (performed in the USA), have measured frustration with technology and its association with EE [44, 45] and included all healthcare professions, and all types of HIT. The association between frustration with HIT and EE was moderate, suggesting the need for further research from different contexts. In our study, we added confounding factors, job demands and resources, and outcomes related to care quality. From an organizational perspective, it is important to detect early signs of stress and exhaustion in healthcare staff as technology continues to transform healthcare workplaces. If technology frustration and stress are identified and addressed, the potential negative influences on job performance [28, 32, 37, 40], patient safety [35, 42, 50–52], and turnover intentions [23, 40, 42, 53–55] can hopefully be avoided. To identify this, there is a need for work environment surveys. Extensive and time-consuming work environment surveys for healthcare staff are preferred by neither management nor staff. Thus, we were interested in using a single-item measure of technology frustration and study associations with staff-rated stress, emotional exhaustion, and satisfaction with given care.

## Method

### Aim

Using JD-R theory as a theoretical framework in a healthcare setting, the aim was to investigate associations between healthcare staff's “frustration with technology” and the three outcomes staff-rated stress, EE, and satisfaction with given care with job resources and the demand workload held constant.

### Design

The study is the first part of a prospective study where data collection takes place before and after changes in the healthcare staff’s digital work environment (to be presented elsewhere). The present study uses data from the premeasurement and thus a cross-sectional correlational design. The data for this study were collected through a survey.

### Sample and setting

This study was conducted in a region in northeast Sweden, where approximately 6 300 of the inhabitants were employed in healthcare. A convenience sample of healthcare staff (n = 1 364) from both public and private clinics in the region was invited to participate. The inclusion criterion was staff with direct patient-related work, regardless of education level. The exclusion criteria were administrative or managing staff, staff absent due to sickness, parental leave or studies, staff close to retirement, and being a substitute. Staff close to retirement were excluded solely because of a prospective study design, where this was the first data collection. Participation was confidential and voluntary.

At the time of the survey, several parallel HIT systems were used within and between clinics in the studied region. The HIT systems were an integral part of daily work but lacked integration with each other, generating excess administrative tasks for all professions. For instance, different regional and national digital platforms were used to prescribe and administer medication. There were at least four different EHR software programs in the region that did not share healthcare data. Furthermore, patient bookings and billings were made from other software programs, not integrated with the EHRs. Most of the laboratory tests and medical imaging were digitalized, but the results were not integrated into all of the coexisting EHRs. The referrals were both digital and on paper. Healthcare data that were not automatically integrated into the EHR needed to be printed and scanned, generating excess administration. Staff communication about a patient could take place within the patient’s EHR, but if the staff worked in different EHR systems, messages weren’t automatically visible to the other. Management communication with staff took place via internal websites and email, and staff working hours and absences had to be manually entered by the staff into a digital platform connected to digital salary payments. Staff meetings were held both in person and via platforms such as Skype, Zoom, or Teams. Patient meetings and assessments were for the most part performed physically in person. Patients contacted healthcare providers by phoning an on-call nurse or visiting their general practitioner or open clinic during office hours and the emergency room at odd hours; patients rarely consulted healthcare via video, and if they did, it was mostly at the caregiver’s initiative.

### Recruitment

In total, 31 head managers in the region were contacted and asked to submit lists of eligible participants, according to the inclusion and exclusion criteria, if they allowed time for their staff to participate in the study. Seventeen head managers responded positively and submitted lists of eligible participants. E-mails containing study information and a link to the survey, were sent to each person on the lists from January to May 2022. Two reminders were sent via e-mail to nonresponders.

### Instruments

To measure study outcomes, we used the dimension stress from the Copenhagen Psychosocial Questionnaire (COPSOQ) version III [56, 57], the EE scale from the Safety, Communication, Operational Reliability, and Engagement questionnaire (SCORE) [58], and the staff satisfaction with given care (SSC) scale [59] (Table 1). The SSC scale was developed in Sweden and is not as commonly used as, e.g., COPSOQ, which makes a short description necessary. The SSC scale is a self-report of the quality of care you have given the patients and thereby also a self-report of healthcare staff performance. The scale has eight items with the heading “How satisfied are you with…” followed by estimations of emotional support “the emotional commitment you showed the patients”, medical information and treatments, availability, and attention to the patient’s well-being. The scale has previously been found to have significant associations with common job resources [33].

**Table 1:** Description of the variables and corresponding instruments used in the study

Variables	Demand or resource	Items	Scale range		Mean (SD)	Median (Q1-Q3)	α
			**Min**	**Max**			
**Outcomes**							
Stress^a,b^		3	1	5	3.3 (0.8)	3.3 (2.8–4.0)	0.85
Emotional exhaustion^c^		5	1	5	2.3 (1.0)	2.2 (1.4–3.0)	0.90
Satisfaction with care^d^		8	1	7	5.8 (0.8)	5.9 (5.3–6.4)	0.91
**Independent variables**							
Improvement readiness^c^	Resource	5	1	5	3.6 (0.8)	3.8 (3.2–4.2)	0.90
Teamwork climate^c^	Resource	7	1	5	3.8 (0.7)	3.9 (3.3–4.4)	0.81
Growth opportunities^c^	Resource	6	1	5	3.9 (0.8)	4.0 (3.5–4.5)	0.90
Participation in decision-making^c^	Resource	6	1	5	3.6 (0.8)	3.7 (3.2–4.2)	0.86
Sense of community at work^a,b^	Resource	3	1	5	1.8 (0.7)	1.7 (1.3–2.0)	0.84
Workload^c^	Demand	5	1	5	3.8 (0.8)	3.9 (3.4–4.4)	0.86
Frustration with technology^c,e^	Demand	1	1	4	1.9 (0.9)	2.0 (1.0–3.0)	

^a^High mean values indicate low stress symptoms and a low sense of community at work, respectively

^b^COPSOQiii. A 5-point Likert scale. (1 = “always”, 5 = “never/hardly ever”)

^c^SCORE. A 5-point Likert scale. (1 = “disagree strongly”, 5 = “agree strongly”)

^d^SSC. A 7-point Likert scale. (1 = “not at all”, 7 = “to a very high degree”)

^e^Single item from SCORE. A 4-point Likert scale. (1 = “rarely or none of the time”, 4 = “all of the time”)

The independent variables were also chosen from SCORE and COPSOQ. To measure feelings of frustration with technology in the workplace, we used a single item from the work-life climate scale in SCORE: “During the past week, how often did this occur? Felt frustrated by technology”, with options ranging from “rarely or none of the time” to “all of the time” on a 4-point Likert scale. This item has been previously studied in association with work-life climate and EE [45], allowing us to compare results. Table 1 describes all the variables and response scales. All the variables have been shown to have good validity and reliability (all with Cronbach’s alpha (α) values > 0.70) in earlier studies.

### Data analysis

The data were analyzed using IBM SPSS Statistics, version 27.0.1 (SPSS Inc., Chicago, IL, USA). Nonparametric statistics were used for univariate and bivariate analyses, as several variables were not normally distributed. Differences between groups (sex, education) and the three outcomes were tested with the Mann–Whitney U and Kruskal–Wallis tests. Bivariate associations were tested with the Spearman correlation coefficient. For multiple regression analyses, an adequate sample size was calculated with the formula N ≥ 50 + 8* m* (*m* = number of independent variables), indicating that we had a sufficient number of cases in the present study [60]. For the multiple regressions, generalized estimating equations (GEE) were used to adjust for possible clustering effects within clinics [61]. Variables with a *p*-value ≤ 0.1 in the univariate and bivariate analyses were included in the GEE models. For cross-sectional and clustered data, exchangeable working correlation matrixes are recommended [61, 62]. However, we also tested the independent and unstructured correlation matrixes [63] but found the best model of fit in the exchangeable working correlation matrix, which was subsequently used for the data analysis. The GEE residuals showed no serious deviations from the normal distribution. The Variance Inflation Factor (VIF) values from linear regression analyses were all less than 2.9, indicating a low risk for multicollinearity [64, 65]. The internal consistency was α > 0.80 for all the variables (Table 1). The significance level was set to *p* ≤ 0.05 in the GEE analyses and 95% confidence intervals were used to indicate the precision of the estimates.

## Results

Five of 12 eligible primary care clinics (2 of 7 private and 3 of 5 public) and 12 of 19 public specialist care clinics participated in the study. Some clinics declined due to a heavy workload or because the head managers did not feel the survey was suitable, and some did not reply. In total, 1 364 healthcare staff at 17 clinics were invited to participate, and 417 responded, yielding a response rate of 31%. The responders were from a wide range of professions (for example, assistant nurses, registered nurses, specialist nurses, occupational therapists, social workers, psychologists, and physicians with different degrees of specialization and experience). Some professional groups, i.e., occupational therapists, were too small for valid analysis (Table 2). Since there is evidence that education level is associated with work stress outcomes [66, 67], we grouped the responders by educational level. The characteristics of each group (sex, education level, and age) are presented in Table 3, and below the table, we refer to all professions included in the study.

**Table 2:** Characteristics of the study participants by profession/role and workplace

	*n (%)* *N* = *417*	*n in type of work* *Primary care/Specialist care*
**Assistant nurses/licensed practical nurses**	**45(10.8)**	**9/36**
**Registered nurses**	**72 (17.3)**	**18/54**
**Specialist nurses**	**102 (24.5)**	**43/59**
by specialty:		
Community Health	23	22/1
Diabetes	8	6/2
Midwifery	21	8/13
Oncology	13	0/13
Ophthalmology	6	0/6
Osteoporosis	2	2/0
Pediatrics	2	2/0
Others^a^	5	0/5
**Occupational therapists**	**4 (1.0)**	**3/4**
**Social workers**	**29 (7.0)**	**16/13**
**Psychologists**	**19 (4.6)**	**5/14**
**Physiotherapists**	**25 (6.0)**	**22/3**
**Physician, intern**	**2 (0.5)**	**2/0**
**Physician, resident**	**36 (8.6)**	**8/28**
by specialty:		
Child and adolescent psychiatry	1	0/1
Family medicine	8	8/0
Internal medicine	1	0/1
Obstetrics and Gynecology	5	0/5
Orthopedics	3	0/3
Oncology	1	0/1
Ophthalmology	4	0/4
Oto-Rhino-Laryngology	6	0/6
Psychiatry	2	0/2
Surgery	5	0/5
**Physician, senior specialist**	**65 (15.6)**	**19/46**
by specialty:		
Family medicine	19	16/3
Geriatric medicine	1	0/1
Internal medicine	3	2/1
Obstetrics and Gynecology	6	0/6
Orthopedics	4	0/4
Oncology	4	0/4
Ophthalmology	6	0/6
Oto-Rhino-Laryngology	2	0/2
Palliative medicine	2	0/2
Psychiatry	3	0/3
Surgery, including plastic- and vascular subspecialties	7	0/7
Urology	4	0/4
Unspecified	4	1/3
**Others, by profession or role**^b^	**18 (4.3)**	**5/13**
Audiologist/hearing aid specialist	1	0/1
Hearing aid engineer	1	0/1
Optician	3	0/3
Psychotherapist	3	0/3
Rehabilitation Coordinator	7	5/2
Special Educated Teachers (of visual or hearing impairments)	3	0/3

^a^Nephrology, neurology, medical devices specialist, surgery, and one unspecified

^b^The education level in this group varied from 3 to > 4.5 years of higher education

**Table 3:** Characteristics of the study participants and staff in the participating and invited clinics

	*n (%)* *Participants* *n* = *417*	*N (%)* *Participating clinics N* = *2029*	*N total (%)* *Invited clinics* *N total* = *5084*
**Sex**			
Female	331 (79.4)	1 731 (85.3)	4 171 (82.0)
Male	83 (19.9)	298 (14.7)	913 (18.0)
No answer	3 (0.7)	-	-
**Education**^**a**^** within healthcare**			
0 < 3 years of higher education	45 (10.8)	452 (22.3)	1 253 (24.6)
3 – 4.5 years of higher education	156 (37.4)	776 (38.2)	1 950 (38.4)
> 4.5 years of higher education	216 (51.8)	801 (39.5)	1881 (37.0)
**Primary care, staff in 5 clinics**^**b**^	150 (36.0)	607 (29.9)	939 (18.5)
**Specialist care, staff in 12 clinics**^**c**^	267 (64.0)	1 422 (70.1)	4 145 (81.5)
**Age**	Range 21–72 years M 45.8, SD 11.3 Md 46	Range 19–68 years Mean 44.1, SD 12.1 Md 43	Range 19–68 years Mean 44.1, SD 12.0 Md 44
< 20	0 (0.0)	1 (0.1)	1 (0.0)
20–29	31 (7.4)	248 (12.2)	632 (12.4)
30–39	107 (25.7)	569 (28.0)	1 414 (27.8)
40–49	113 (27.1)	481 (23.7)	1 204 (23.7)
50–59	102 (24.5)	453 (22.3)	1 165 (22.9)
> 60	64 (15.3)	277 (13.7)	668 (13.1)

^a^ The 0- < 3-year group mainly consisted of licensed practical nurses or assistant nurses. The 3–4.5-year group consisted of registered nurses, social workers, occupational therapists, physiotherapists, audiologists, hearing aid engineers, and opticians. The > 4.5-year group consisted of physicians, psychologists, specialist nurses, psychotherapists, and special educated teachers

^b^ Clinics within primary care included general healthcare clinics and psychosocial- and rehabilitation teams

^c^ Clinics included within specialist care were surgery, internal medicine, orthopedics, oncology, ophthalmology, oto-rhino-laryngology, psychiatry, child and adolescent psychiatry, gynecology, and obstetrics

### Bivariate correlations and differences between groups

Statistically significant associations were found between all the independent variables and the outcomes, except between workload and SSC (Table 4). The variable frustration with technology was associated with stress, EE (both *p* < 0.001), and SSC (*p* = 0.01). Increasing age was significant for all the outcomes (stress *p* < 0.001, SSC *p* < 0.001, EE *p* = 0.045).

**Table 4:** Bivariate associations between study variables with Spearman’s rank correlation coefficient

**Outcomes**	**Mean (SD)**		**Improvement readiness**^**d**^	**Teamwork climate**^**d**^	**Growth opportunities**^**d**^	**Participation in decision-making**^**d**^	**Sense of community** **at work**^**a**^	**Workload**^**e**^	**Age**	**Frustration with technology**^**e**^
**Stress**^**a**^	3.3 (0.8)	Correlation coefficient	.274	.366	.357	.382	-.373	-.424	.207	-.281
		n	413	415	415	416	414	416	416	414
		Sig. (2-tailed)	** < .001**	** < .001**	** < .001**	** < .001**	** < .001**	** < .001**	** < .001**	** < .001**
**Emotional exhaustion**^**b**^	2.3 (1.0)	Correlation coefficient	-.402	-.500	-.527	-.452	.435	.572	-.099	.326
		n	414	416	416	416	414	415	416	414
		Sig. (2-tailed)	** < .001**	** < .001**	** < .001**	** < .001**	** < .001**	** < .001**	**.045**	** < .001**
**Staff satisfaction with care (SSC)**^**c**^	5.8 (0.7)	Correlation coefficient	.249	.309	.317	.335	-.319	-.027	.173	-.161
		n	412	414	414	415	413	415	415	413
		Sig. (2-tailed)	** < .001**	** < .001**	** < .001**	** < .001**	** < .001**	.578	** < .001**	**0.01**

^a^ High values for the variables stress and sense of community at work indicate low stress symptoms and a low sense of community at work, respectively

^b^ High values of the emotional exhaustion variable indicate high emotional exhaustion symptoms

^c^ High SSC values indicate high satisfaction with given care

^d^ High values for improvement readiness, teamwork climate, growth opportunities, and participation in decision-making indicate a positive rating of the variable in the workplace

^e^ High values for workload and frustration with technology indicate a high rating of the burden of work and a frequent feeling of frustration with technology

Bold numbers indicate statistically significant values

The statistics for the differences between the groups are presented in Table 5. Stress differed significantly between the sexes (*p* = 0.016). For the other outcomes, the associations with sex were nonsignificant (EE *p* = 0.138, SSC *p* = 0.907). Educational level was nonsignificant for all outcomes (stress *p* = 0.511, EE *p* = 0.889, SSC *p* = 0.898) and was therefore excluded from further analysis.

**Table 5:** Outcomes compared between males and females and different educational levels

	**Stress**			**EE**			**SSC**		
	n	p	Mean (SD)	n	p	Mean (SD)	n	p	Mean (SD)
			3.3 (0.8)			2.3 (1.0)			5.8 (0.7)
**Sex**^a^		.016			.138			.907	
Male	83		3.5 (0.9)	83		2.2 (1.0)	82		5.8 (0.8)
Female	330		3.3 (0.8)	330		2.4 (1.0)	330		5.8 (0.7)
**Educational level**^b^		.511			.889			.898	
0 < 3 years	44		3.3 (0.9)	45		2.3 (1.0)	44		5.9 (0.8)
3 – 4.5 years	156		3.3 (0.8)	155		2.3 (1.1)	155		5.8 (0.8)
> 4.5 years	216		3.4 (0.8)	216		2.3 (1.0)	216		5.8 (0.7)

^a^Mann-Whitney U test

^b^Kruskal Wallis test

High values in the stress variable indicate low stress symptoms

High values in the emotional exhaustion variable indicate high emotional exhaustion symptoms

High values in the SSC variable indicate high satisfaction with given care

#### GEE models

Two separate GEE analyses were performed for each outcome, one without and one with the variable “frustration with technology” included in the analysis (Model 1 [M1] and Model 2 [M2], respectively, Table 6).

**Table 6:** GEE models of outcomes without and with the variable frustration with technology

**Variables**	**Stress**^a^ (scale range 1-5)				**Emotional exhaustion** (scale range 1-5)				**Staff satisfaction with care** (scale range 1-7)			
	**B**	***p***-value	**95% Confidence Interval**		**B**	***p***-value	**95% Confidence Interval**		**B**	***p***-value	**95% Confidence Interval**	
			Lower	Upper			Lower	Upper			Lower	Upper
**Model 1**												
Improvement readiness	-.102	.156	-.244	.039	-.042	.518	-.171	.086	-.060	.331	-.182	.061
Teamwork climate	.102	.147	-.036	.241	-.248	**<.001**	-.378	-.118	.088	.298	-.078	.254
Growth opportunities	.041	.534	-.089	.171	-.350	**<.001**	-.439	-.262	.071	.344	-.076	.219
Participation in decision-making	.243	**.002**	.085	.401	-.006	.946	-.184	.172	.247	**<.001**	.145	.350
Sense of community at work^a^	-.200	**.008**	-.348	-.053	.121	.128	-.035	.276	-.040	.623	-.198	.119
Workload	-.369	**<.001**	-.491	-.248	.549	**<.001**	.421	.677	-	-	-	-
Age	.014	**<.001**	.007	.021	-.007	.079	-.014	.001	.010	**<.001**	.006	.014
Sex	.201	**.012**	.045	.357	-	-	-	-	-	-	-	-
**Model 2**												
Improvement readiness	-.116	.131	-.268	.035	-.033	.627	-.164	.099	-.067	.298	-.193	.059
Teamwork climate	.083	.241	-.056	.222	-.234	**<.001**	-.369	-.099	.078	.339	-.082	.238
Growth opportunities	.042	.523	-.087	.172	-.349	**<.001**	-.434	-.263	.070	.348	-.077	.217
Participation in decision-making	.222	**.006**	.063	.381	.001	.991	-.177	.179	.242	**<.001**	.133	.351
Sense of community at work^a^	-.201	**.007**	-.346	-.056	.120	.133	-.037	.276	-.037	.645	-.196	.122
Workload	-.334	**<.001**	-.443	-.225	.529	**<.001**	.417	.641	-	-	-	-
Age	.014	**<.001**	.008	.021	-.007	.076	-.014	.001	.010	**<.001**	.006	.014
Sex	.271	**<.001**	.116	.426	-	-	-	-	-	-	-	-
Frustration with technology	-.154	**<.001**	-.207	-.101	.097	**.019**	.016	.178	-.059	.173	-.144	.026
**VIF**^b^												
Max	2.833 (Participation in decision-making)				2.801 (Participation in decision-making)				2.803(Participation in decision-making)			

^a^Note that high mean values for these variables indicate low stress symptoms and a low sense of community at work, respectively

^b^VIF = Variance inflation factor from regression analyses including frustration with technology

Hyphen = the variable was not included in the analysis

Bold numbers indicate statistically significant values

##### Stress

Model 1 revealed statistically significant associations between lower stress (higher scores on the scale) and higher participation in decision-making, a higher sense of community at work, lower workload, male sex, and increasing age. In Model 2, the variable frustration with technology was added, which did not lead to any changes in the statistically significant associations in Model 1 but added a statistically significant association between higher stress and higher frustration with technology.

##### Emotional exhaustion

In Model 1, higher EE was statistically significantly associated with a lower teamwork climate, lower growth opportunities, and higher workload. In Model 2, the variable frustration with technology was added, which did not lead to any changes in the statistically significant associations in Model 1 but added a statistically significant association between higher EE and higher frustration with technology.

##### Staff satisfaction with given care

In Models 1 and 2, higher SSC was statistically significantly associated with higher participation in decision-making and increasing age. The variable frustration with technology added in Model 2 was nonsignificant.

##### Frustration with technology

In summary, the addition of the variable frustration with technology in the second GEE model revealed significant associations with stress (p = < 0.001) and EE (p = 0.019) but not with SSC (Model 2, Table 6). In the sample, 26.4% reported that they felt frustrated by technology “occasionally or a moderate amount of time” (3) or “all of the time” (4) on a 4-point Likert scale.

## Discussion

This study aimed to investigate whether healthcare staff’s feelings of frustration with technology are associated with stress, emotional exhaustion, and satisfaction with given care, with JD-R theory as the guiding framework. The results showed that frustration with technology, measured as a single item, was associated with elevated levels of stress and EE for healthcare staff when the demand workload and other common job resources were held constant, and potentially confounding factors such as age and sex were controlled for. Our results in the GEE models are consistent with previous research on EE [44, 45] and burnout [1, 10–12]. This could imply that frustration with technology is a sign of high HIT demands and that HIT demands are associated with stress and emotional exhaustion, which is consistent with previous research [18, 19] and with JD-R theory [28].

In this study, frustration with technology was rated moderate to high by 26.4% of the staff, similar to the results by Tawfik et al. [45] (32.7%). The technology frustration in our sample is better understood when reviewing the work context: staff were required to manage multiple HIT systems simultaneously, with a lack of interoperability leading to excess administration and thus impeding job performance [68]. In addition, the clinics used different EHR systems, which impeded readability, coherence, and written communication across the systems, possibly leading to uncertainty [68] about the quality and safety of the provided care. Research has shown that working with HIT alone can lead to psychological distress [69] and that a high technological workload (techno-overload, information overload) increases symptoms of exhaustion and burnout [70]. Our bivariate correlation results suggested alignment with these findings, as frustration with technology was significantly associated with higher estimates of stress and emotional exhaustion. The strength of the associations was moderate [71] for stress and EE (rho -0.281 and 0.326, respectively).

In the bivariate analysis, technology frustration was also associated with lower estimates of SSC, but the association was weak [71] (rho -0.161). In the GEE model, this association changed from significant to nonsignificant. Interestingly, the associations between high workload and lower estimates of SSC were also nonsignificant. This contradicts previous research [59, 72] that has reported associations between workload and staff-assessed quality of care. One possible explanation for these findings could be that healthcare staff strive to provide patients with good care, regardless of workload or frustration. Since there were statistically significant and moderate associations between all the job resources and SSC in the bivariate analysis, it could also be congruent with the JD-R assumption that resources can facilitate job performance despite a high workload [28, 29, 31].

For the other outcomes in the GEE models estimated here, significant associations for stress were observed with participation in decision-making, a sense of community at work, workload, sex, and age. EE was significantly associated with teamwork climate, growth opportunities, and workload. SSC was significantly associated with participation in decision-making and age. These results are in line with JD-R theory and with earlier research [42, 54, 69, 73–77]. Workload (significant for stress and EE outcomes) and participation in decision-making (significant for stress and SSC outcomes) were the two most recurring significant variables in the GEE models. It is well documented that a high workload contributes to stress and EE [29, 30, 32, 42, 74]; thus, the significant associations we found in our survey were expected. Our findings that participation in decision-making and having a high sense of community (i.e., social capital) are associated with lower stress are in line with previous research [72, 77, 78]. However, these resources have also been associated with lower EE [78, 79], which we also found in the bivariate analysis but not in the GEE analysis. In line with previous research, our findings revealed that a low teamwork climate [42, 44, 75, 80] and low growth opportunities [80, 81] were associated with EE.

The fact that feelings of frustration with technology are associated with higher EE, is important to address further considering that high EE among healthcare staff is also associated with higher intentions to leave [22, 23, 42, 53–55], decreased patient safety [35, 42, 50–52] and lower care quality [22, 23, 42].

The causes of technology frustration were not investigated in this study, but previous research has found possible triggers in technology-related demands such as poor usability [5, 17, 68, 82] and pressure to learn new skills [20, 49, 68, 83]. Not being part of the developmental or implementation process for a new HIT system has also been associated with frustration among healthcare staff [3, 68, 84]. When feelings of frustration with HIT occur in staff, it should be seen as a signal of underlying technological demands that need to be defined and resolved. Future studies should explore such demands and associations with outcomes related to staff well-being and performance.

### Strengths and limitations

This study has several limitations. First, the cross-sectional design limits conclusions about causality. Second, the questionnaire included more resources than demands, which can skew the interpretation of the findings. However, all the questions were from validated instruments, and robust statistical methods were used to analyze the data, reducing response bias [85] and measurement errors.

Using a convenience sample from only one healthcare region limits the generalizability of the results. However, a strength was the diverse study population, which included all healthcare professionals from direct patient-related work. A skewness in the sample compared with the group as a whole was observed for education level (Table 3). Responders with educational levels < 3 years of university studies had a lower response rate than the total eligible group within the region, and responders with educational levels > 4.5 years of university studies had a higher response rate than the total eligible group within the region. Assuming that the number of different HIT systems to handle in everyday work increases with higher educational levels, this skewness might threaten the validity of the results. The low response rate overall and convenience sample also increase the risk of several sampling biases, impacting interpretation and limiting the generalizability of the results. For example, some clinics declined to participate because of heavy workloads, which, coupled with a potential nonresponse bias from staff with the highest workload, entails a substantial risk that staff with the highest job demands were excluded from the study. Another nonresponse bias might have been general fatigue due to high stress after the COVID-19 pandemic when Swedish healthcare regions strived to catch up with the care backlog caused by the pandemic, or perhaps “survey fatigue”. Negative affectivity traits [86, 87] might also have skewed some respondents’ estimates negatively but might also have led to decreased participation. A potential response bias might be that those who found time to answer the survey were staff with a lighter workload and thus lower job demands, which can skew the results. However, the low response rate was considered acceptable and valid [88, 89] given the method of a voluntary online survey to a convenience sample in healthcare, without financial incentives [90], and the risk of inadequate participant lists. The large and diverse sample may have reduced the overall response/nonresponse bias risk.

### Practical implications and further research

As described at the beginning of this article, technology can be either a demand or a resource in a workplace [21, 91]. In further research on technology’s role in healthcare, it is important to investigate what HIT demands lead to frustration and what generates or mediates negative or positive health outcomes. Furthermore, objective variables such as measuring system response time (SRT) in HIT systems could be included to increase the validity and generalizability of the results, as well as to investigate causality with stress and EE. Future studies should also aim to apply longitudinal designs to investigate possible causalities between digital transformation and work health. This study shows that one single-item question can be used as a gauge of staff well-being in association with HIT. This result is relevant for further research but also of practical importance for healthcare managers tasked with evaluating staff’s psychosocial work environment. Based on this study, we suggest that established questionnaires for assessing staff working life should be augmented with questions about technology alongside other job demands and resources.

## Conclusions

This study adds new findings that feelings of frustration with technology in healthcare are associated with moderately elevated ratings of stress and emotional exhaustion, although not with satisfaction with given care. However, further studies are needed to confirm these results and to investigate what causes technology frustration in healthcare staff and how to mitigate it before it contributes to stress and exhaustion.

## Data Availability

Complete datasets from the current study are not publicly available due to privacy law/general data protection regulation (GDPR) in Sweden. Aggregated data are available from the corresponding author upon reasonable request.

## Abbreviations

COPSOQ: The Copenhagen Psychosocial Questionnaire
EE: Emotional Exhaustion
EHR: Electronic health records
HIT: Health information technology
JD-R: Job Demands-Resources
SCORE: Safety, Communication, Operational Reliability, and Engagement questionnaire
SRT: System Response Time
SSC: Staff satisfaction with care
